# Mechanisms of Techno-Moral Change: A Taxonomy and Overview

**DOI:** 10.1007/s10677-023-10397-x

**Published:** 2023-06-01

**Authors:** John Danaher, Henrik Skaug Sætra

**Affiliations:** 1 School of Law, NUI Galway, University Road, Galway, Ireland; 2 Ostfold University College, Norway, Ireland

**Keywords:** Technology, Moral change, Ethics, Engineering ethics, Artificial intelligence, Digital technology, Robotics

## Abstract

The idea that technologies can change moral beliefs and practices is an old one. But how, exactly, does this happen? This paper builds on an emerging field of inquiry by developing a synoptic taxonomy of the mechanisms of techno-moral change. It argues that technology affects moral beliefs and practices in three main domains: *decisional* (how we make morally loaded decisions), *relational* (how we relate to others) and *perceptual* (how we perceive situations). It argues that across these three domains there are six primary mechanisms of techno-moral change: (i) adding options; (ii) changing decision-making costs; (iii) enabling new relationships; (iv) changing the burdens and expectations within relationships; (v) changing the balance of power in relationships; and (vi) changing perception (information, mental models and metaphors). The paper also discusses the layered, interactive and second-order effects of these mechanisms.

## Introduction

It has long been noted that technology can result in changes in social morality (White 1962; Greenwood 2020). To take just one example, new technologies have always challenged and moulded the concept of privacy. Westin, writing in the late 1960s, for example, was concerned with how new technologies in his time challenged the value of privacy and normalised surveillance (Westin 1967). With the emergence of the internet, mass surveillance, and digital convenience, people’s notions and valuation of privacy and its related rights and duties have been challenged and are arguably in flux (Hartzog 2018; Debrabander 2020; Zuboff 2019). Recent scholarship has explored the relationship between technology and moral change in some detail. Much of this scholarship focuses on individual case studies (Nickel 2020; Baker 2019; Kudina and Verbeek 2019, Swiestra et al. 2009; Eriksen 2020; Danaher 2021; Nyholm 2021). Some of it is more theoretical in nature, identifying general patterns of moral and social change (Pleasants 2018; Baker 2019, Perez 2003; Morris 2015) or suggesting some possible mechanisms of moral change or ‘mediation’ (e.g. Verbeek 2011; 2013; Kudina and Verbeek 2019; Danaher 2021; van de Poel and Kudina 2022; Hopster et al. 2022). Other contributions are more normative, focusing on the moral and practical implications of technologically-mediated moral change (Poel 2021; Nickel 2020).

This paper contributes to this growing literature on technology and moral change by providing a synoptic account of what we take to be the key mechanisms through which technology alters moral beliefs and practices. The article describes six specific mechanisms through which this happens, and then considers their layered, interactive and second-order effects. While we do not claim that the taxonomy is exhaustive, we do believe that it captures the primary mechanisms through which technology can effect moral change. The taxonomy is, consequently, something that can provide a basis for future scholarship and inquiry into this important issue. The paper starts with a brief overview of the topic, clarifying its point of focus and explaining why it is worthwhile providing a synoptic account of the mechanisms of technologically mediated moral change. The bulk of the paper is then taken up with a detailed description of the six mechanisms. It concludes by explaining how these mechanisms might interact and give rise to second-order moral effects, which provides an enriched and more complex account of the role of technology in moral change.

## The Study of Technologically Mediated Moral Change

Technology is, of course, a contested concept. In a review of the history of thinking about technology, Schatzberg (2018) argues that the predominant historical mode of thinking about technology has been an *instrumentalist* one, i.e. technologies are tools that humans use to accomplish goals. They are extensions of ‘means-end’ reasoning, nothing more. He contrasts this with an alternative school of thought, which he prefers, which he calls the *cultural* school of thought. According to this, technology is an expression of artistic or creative agency, not just means-ends reasoning. Related to this, there is a dispute between *materialist* theories of technology, which see technologies as, necessarily, material artifacts (hammers, nuclear bombs, computers and so on), and *institutionalist/idealist* theories, which also include abstract social institutions such as ‘democracy’ or the ‘rule of law’ or the ‘free market’ within the ambit of technology (see Arthur 2009 for a longer discussion of this debate). While we think there is wisdom in adopting a culturalist and institutionalist understanding of technology, for present purposes, we presume a largely materialist-instrumentalist understanding of what technology is. In other words, we assume that technologies are, primarily, material artifacts created by humans to assist with means-ends processes. Some technologies, developed in this manner, may take on a life of their own (quite literally – a possibility we discuss later on) but they begin as material artifacts used to accomplish goals.

Technology, so understood, plays a key role in *mediating* the relationship between humans and the world around them. The philosopher Don Ihde developed one of the classic frameworks for understanding this mediating effect (Ihde 1990). Ihde’s framework was quite general in scope, describing four types of mediating effects that technology can have on how humans relate to the world: *embodiment* (i.e. we extend our bodies through technology), *hermeneutic* (technology changes how we interpret the world), *alterity* (we relate directly to the technology as ‘other’), and *background* (technology becomes part of the background furniture of the world). There have been several innovations on Ihde’s framework over the years. Of particular interest to this paper is Peter Paul Verbeek’s account of the moral mediating effect of technology (Verbeek 2011, 2013). Verbeek’s theory is a complex one, and has been developed and refined by others (e.g. Kudina 2019). The gist of it, however, is captured in the following quote:

“*The central idea [of the technological mediation of morality] is that technologies-in-use help to establish relations between human beings and their environment. In these relations, technologies are not merely silent ‘intermediaries’ but active ‘mediators’ … By organizing relations between humans and world, technologies play an active, though not a final, role in morality. Technologies are morally charged, so to speak. They embody a material form of morality, and when used, the coupling of this ‘material morality’ and human moral agency results in a ‘composite’ moral agency.”*(Verbeek 2013, pp 77–78).

How is it that technology plays this “active, though not final role” in morality? Verbeek has tried to clarify the distinct forms of technological mediation in his work, noting in particular that technology changes how we make morally charged decisions, and how we interpret or understanding morally charged phenomena (we discuss specific examples from Verbeek’s work later in this article). Furthermore, although Verbeek articulates his theory using the concept of ‘mediation’, the core of his analysis is not particularly novel. One can find variations on it in the earlier work of, for example, Langdon Winner with his claim that technologies can have ‘politics’ (Winner 1977).

In addition to this work on the mediating effect of technology, there is an active research literature focusing on the role of technology in social and moral change. There are too many sources to list here, but some prominent examples include the work of Tsjalling Swierstra on technomoral change (2009, 2013), Ibo van de Poel on responsible innovation and value change **(**van de Poel 2021; van de Poel and Kudina 2022), Ian Morris on technologies of energy capture and value systems (Morris 2015), Stephen Barley on technological changes to workplace norms and power relations (Barley 2020), Philip Nickel on technologies and moral disruption (Nickel 2020), and many more. What each of these discussions shares is a common belief that technology plays a key role in changing how humans perceive moral values and make moral choices. As we have noted, the specific focus on morality as a concept is somewhat new, but the modes of analysis and the topics examined mirror earlier attempts to understanding the social shaping power of technology by authors such as Mumford (1934), Ellul (1964), and Langdon Winner (Winner 1977).

Some of the work done to date has been quite abstract and general, focusing on largescale impacts of technology on social value systems (e.g. Morris 2015), some is more specific, focusing on specific case studies in technology and moral change (e.g. Swierstra 2013), some tries to extract general lessons from specific case studies (Hopster et al. 2022). The goal of this paper is to complement and enhance the work that has been done to date by providing a synoptic account of the primary mechanisms through which technology can change human morality. In presenting this account, we follow Verbeek in not presuming that technology plays a decisive role in changing human morality. Ideologies, cultural institutions, as well as environmental and material constraints, play an important role too (cf. Eriksen 2020, Appiah 2010, Baker 2019, Buchanan and Powell 2018; Danaher 2021; Hopster 2022). But technology can play some role in the process and this may, on occasions, be crucial.

But what does it mean to say that technology can change morality? It should be noted that when we talk about technologically mediated changes in human morality, we are referring to changes in how people *perceive* and *understand* the Good (what is worth pursuing, valuing and promoting) and the Right (what is obligatory, permissible, forbidden and so on) and how they act on those perceptions and understandings.^1^ We are not referring to actual changes in what is Good and what is Right. In other words, the moral truth could well be invariant across time and space. We pass no judgment on this. But our moral beliefs and habits do change across time and space. A casual glance at the historical (e.g. Malik 2014, Lecky 1955) and cross-cultural (Flanagan 2017) record reveals this to be the case. It is the impact of technology on these moral beliefs and habits that is our main focus. To put it another way, our focus here is on changes to social morality, not changes to ideal morality (cf. Danaher and Hopster 2022 for a discussion of the normative significance of such changes).

We believe that the account we offer in the remainder of this article is valuable for two main reasons. The first is theoretical and scholarly: by unifying and clarifying what we take to be the primary technological mechanisms of moral change, we believe that we will enhance the scholarly discourse about this topic. In addition to the obvious theoretical virtues of a unified and synoptic account, future researchers will have a framework upon which they can draw when developing case studies in technology and moral change or when choosing research questions. The second reason is more practical. A common plea among researchers in this area is that the designers of technologies need to take the moral effects of technology more seriously (Poel 2021; Kudina and Verbeek 2019). By clarifying the possible mechanisms through which technology might change morality, we can assist the engineers, designers and users of technology in taking this idea seriously. We can also help policy makers and activists that care about the moral impact of technology understand and articulate their concerns. Future researchers may refine and expand upon what we have to offer. We welcome such developments. But having a reasonably abstract and general account of the mechanisms is a useful starting point.

## The Taxonomy of Mechanisms

The remainder of the paper will follow a common format. We will introduce a specific mechanism of change, describe how it works, and give various examples. We will occasionally refine our account of those mechanisms by addressing possible complications and exceptions. After running through six primary mechanisms, we consider how the mechanisms might layer on top of one another in the case of any one technology, and also their interactive and second-order effects (what is meant by this terminology will be clarified later). The paper does not set out to defend any particular thesis about technology and moral change. We do not, for instance, claim that any particular mechanism is more important than another. We simply aim to explain and understand the mechanisms. The end result, we hope, is a useful framework for anyone interested in understanding and addressing the impacts of technology on social morality.

### Mechanism 1—Technology Changes Option Sets

The first technology-driven mechanism of moral change is, perhaps, the most obvious: technology changes option sets. Human life is replete with decisions. In making decisions, we select among options. Do we eat ice-cream or cake? Do we drive to work or walk? Some, maybe even most, of these decisions have a moral dimension. We prefer options that promote our values (increase the good; decrease the bad) and, if we are guided by morality, we try to choose right over wrong. One of the obvious impacts of technology is to introduce new options to our lives—to use a technology to achieve some goal—that were previously unavailable. This can, as Hopster et al. (2022) observe, change the decision-making landscape. That said, it is not always a simple case of adding more options. Adding options for some people can come at the expense options for others. So the net effect of adding technologically mediated options is not straightforward. We discuss this below.

Let’s take a simple example. The creation of the smartphone, with its camera and internet connectivity, along with associated social media apps, adds options to our everyday lives. Where once we had to sit silently with our own thoughts, we now have the option of endless online distraction. Where once we had to live in the moment and enjoy the concert we were attending, now we have the option of capturing it and sharing it with our social media followers. Many other examples could be given. Suffice to say, this is a very common effect of technology.

Does technology always add options? Does it sometimes take options away? There are, famously, some technologies that are designed to take options away from us. Winner (1980), in his famous discussion of whether technologies can have politics, argued that the construction of low-lying bridges on Long Island took away the option of bus travel to public beaches for people that did not own cars (usually people from ethnic minority groups). More recent examples might include the creation of internet blocking software is supposed to take away the option of surfing the web (usually in an effort to increase workplace productivity). Similarly, alcohol interlocks in automobiles take away the option of driving while drunk. But it doesn’t seem quite right to say that these technologies take options away en masse. This is only true if you take a narrow view of decision-making. They may take away old options from some but they typically do so by giving new options to others. For example, option-blocking technologies take away ‘in-the-moment’ options, but do so by adding options upstream of that moment. So, for example, the alcohol interlock takes away the option of driving while drunk, but only if you previously exercised the option to install such a device in your car. It’s true, of course, that other people might impose option-blocking technologies on us (e.g. court-ordered use of the interlock) and this might take away an option from us, but, again, this is only because the technology added that option for someone else. The net effect, in most cases, is that technology increases options, if not always for the individual in the moment, then at least for society as whole across all moments.

This could be argued to be one example of how technology tends to increase the *complication* of decisions in human societies, while not necessarily increasing the underlying complexity of choices. As argued by Næss (1999), complexity in the sense of non-linear, chaotic structure is a fundamental aspect of life and ecology and captures the qualities of relations between organisms and their environments. C*omplication*, in the sense of adding more bits or parts to a phenomenon, on the other hand, is often introduced to our lives by technology, such as when just getting from home to work in a big city involves countless decisions (car or bus or walk? Leave early or late? Etc) and random disturbances (traffic jams, railway line breakdowns, accidents and emergencies) while also being dependent on a wide array of decisions made centrally. With increased complication, we need to make more decisions, as individuals and as a society, in a world with more options. This increased complication is best understood in the aggregate, as a society-wide phenomenon. It can sometimes co-occur with simplification or reduced choice for certain individuals. Hence, technology changes option sets, usually by adding options, but not always – at least not for everyone.

Changing options can have the effect of raising new moral questions and moral dilemmas. With new options we have new ways of attaining our values, new value tradeoffs to consider, and so on. When these questions are resolved, we sometimes generate new moral duties and permissions. A classic example of this is the effect that the creation mechanical ventilation had on the morality of organ donation and the understanding of death. This has been widely discussed in the literature (Nickel 2020; Baker 2013). In essence, by adding the option of keeping someone’s body alive after their brain had ceased functioning, we were able to preserve organs for donation that would previously have been lost. This generated a new moral question: should we be allowed to keep people alive to facilitate such donations? In the end, most ethicists agreed that this was permissible, preferably if there was prior consent. This new moral conclusion was prompted by the new option made possible by the technology. It also led to a new definition and understanding of what it meant to be dead: brain death.

The moral impact of changing options is not easy to predict. Sometimes adding options makes our moral decisions more fraught and complex. Decision-making in times of war is, arguably, more morally loaded and complex with the option of nuclear weapons (or bioweapons or chemical weapons) than it is without. We are no longer making decisions to kill particular enemies in particular combat zones but making decisions with potentially much wider ecological and existential effects. What may once have been a straightforward, albeit tragic and high stakes decision, becomes even more high stakes and morally complex with the addition of new, downstream consequences. That said, changing options doesn’t always make things more morally complex and fraught. Sometimes adding options can make moral decision-making more straightforward by reducing the need for moral tradeoffs. Before the advent of the automobile or effective public transport, I may have had to choose between two equally valuable options: attend friend 1’s birthday party or friend 2’s retirement bash. With that technology, I may no longer need to choose between those options: I can have a little of both. I may even oblige myself to attend both functions by making a promise to both friends. This highlights an important point. Even in cases in which technology reduces the need to tradeoff between valuable options, there can be a subtle complicating effect: we have more capacity to achieve morally valuable ends and hence we no longer have as many excuses as we once had. We are forced to moralise certain choices that we could previously have said were beyond our control. Consider, as an example, how wired telephones provided people the option of being unavailable when out of their houses, while cellular phones in effect removes such an option and forces people to defend and explain themselves if they still *choose* to be inaccessible when they need not be.

One important moral effect of adding options is the effect on the value of freedom. It might seem like adding options always has a positive impact on freedom because more options equals more freedom of choice. But, of course, it is not so simple. Sometimes more options undermine our ability to rationally select among those options. We become overwhelmed by the choices. As Cass Sunstein notes, “[i]n many areas, what the choice-making muscle needs is rest, not exercise” (Sunstein 2016a, p. 61[2] ). In fact, the removal of choice, or introduction of choice alleviation, has been a central feature in the nudge literature (Thaler and Sunstein 2003[3] ). This would imply that some might see a technology-driven increase in options as a bad thing, at least if each individual has to choose how to make use of the technology for themselves. However, the negative effect of this increase in choices could be moderated if technology could be used (by others or by oneself) to limit choices, remove previous options, or to assist choices, through, for example, algorithmic decision-support systems or nudging technologies that help through foregrounding the options most likely to be good for us. This is a central idea in the more recent work by Sunstein (2022), as also crystalised in his recent co-authored book *Noise* (Kahneman et al. 2022).We have here, then, a way in which technology might be taken to justify an interventionist and paternalistic approach to social governance: take away the complications of decision-making through increased technological control. Technology has politics, to parrot Winner.

Relatedly, Carter (1999) operates with the term *overall freedom* to describe how free we really are, and this, he argues is discovered by counting all hypothetically available options against those that are actually available to us. If a brand new option is introduced and becomes available, overall freedom increases. If, however, a new option merely replaces a previously available option that becomes unavailable, we are no freer than before. So when assessing the moral impact of technology on the value of freedom, we need to consider the aggregate effect on options, not the individual or specific effect. Alternatively, a perfectionist approach to freedom, such as that advocated by Raz (1986) suggests that what matters to freedom is not the total or aggregate number of choices but, rather, their quality: some options and alternatives are simply more valuable than others (Sætra, 2021c). So whether technology makes us more a less free depends on the quality of the options it gives us, not their sheer quantity.

We don’t wish to become too embroiled in specific applied debates about the ethics of technology. Our crucial point is a more general one, which these specific debates illustrate: one of the primary mechanisms through which technology changes human morality is through the addition of options and the resultant rearrangement of choice sets.

### Mechanism 2—Technology Changes Decision-Making Costs

The second mechanism through which technology can change morality is related to the first: by changing the costs of morally-charged decision-making. ‘Costs’ here must be interpreted broadly to include both the effort and exertion involved in exercising an option, as well as the practical, economic, and moral costs this might entail (costs to values, personal integrity and so forth). By changing costs, technology can make it both harder and easier to (a) access certain values and (b) do the right thing. Another way of putting this is to say that technology changes the availability and ease of access to various options. Berlin (2002) often wrote about options in the form of doors available to us. Connecting to various people around the globe, for example, has become easier with the new technologies that reduce the costs and efforts associated with connection, even if the options themselves might not be, in principle, *new*. However, what matters is not only whether or not a door is available, but also how easily available, perceivable, and openable, it is (Berlin 2002). Technology plays a role in changing the constellation of doors and their ease of access (Sætra and Mills 2022).

Let’s consider an example. One of the most widely discussed case studies of technology and moral change is the impact of cheap and effective contraception on moral attitudes toward extra-marital (primarily pre-marital) sex (Adshade 2013; Greenwood and Guner 2010; Nickel et al. 2022; Hopster et al. 2022). Sex is an important human value. Not all sex is good, of course, but many people desire it and most agree that (good) sex is part of a flourishing human life. Uncontracepted sex carries a risk of unwanted pregnancy and infection. This is one reason—but certainly not the only reason—why sex outside of marriage was, historically, taboo. The advent of cheap and effective forms of contraception changed this. By massively reducing the risk of unwanted pregnancy – from upwards of 85% per year down to less than 5% per year according to some analyses (Greenwood and Guner 2010; Greenwood 2020) – effective contraception changed the decision-making calculus. Once the unwanted effects of extra-marital sex were reduced, the potential benefits (pleasure, sexual intimacy, experiencing the feeling of being desired and wanted) became more available. This has resulted in a sea-change in social moral beliefs and practices. Where once extra-marital sex was shameful and taboo, it is now widely accepted and, in some cases, even celebrated (as a sign of liberation and sexual enlightenment). Indeed, even in countries where dominant religious moral codes continue to condemn it, most people ignore those religious codes and endorse an alternative, more sexually liberated one in their day-to-day lives (Fernández-Villaverde et al. 2014).

In this particular example, technology reduced the negative costs of an action and thereby enabled people to access a value (sexual intimacy) more readily. It doesn’t always work that way. Sometimes technology can increase costs and make it more difficult to access a value. Consider, for example, the impact of social and synthetic media (deepfakes, cheapfakes) on our ability to access the truth. Most people agree that the truth is valuable, either for intrinsic or instrumental reasons (Williams 2002). The exact nature of truth, as well as our beliefs about its nature, are contested (Barnard and Ulatowski 2013, 2019). The classic correspondence theory of truth suggests that truth consists in having beliefs that correspond to reality. In some contexts this maps on well to the ‘folk’ conception of truth, but not in all cases (Barnard and Ulatowski 2013, 2019). More modern theories of truth lean into pragmatism, suggesting that having truth beliefs is about following the epistemic norms associated with different disciplines and practicing good epistemic hygiene, and this may map onto folk beliefs about truth in other domains. Whatever the case may be it is now, arguably, harder to gain true beliefs as a result of digital media technology. For one thing, the algorithmic curation of information on platforms such as Facebook and Twitter means people tend to be exposed to information that confirms their pre-existing biases and leaves them trapped in a certain worldview, without the ability or incentive to correct any errors they might have or follow sound epistemic norms (Sætra, 2021). For another thing, the sheer volume of information, in combination with the rise of hyperrealistic fake information, makes it harder to sort fact from fiction (Fallis 2021; Rini 2020). This makes the truth harder to work out and more difficult to obtain. This has proven to be a particular problem with respect to politically or socially contentious issues, as is clear from the widespread misinformation shared and disseminated during the COVID-19 pandemic.

This can have a number of further moral effects. First, by making the truth more difficult to obtain, we might be encouraged to seek out other related values instead, i.e. to substitute the costly, difficult-to-obtain value of truth for another cheaper substitute value. If it is hard to find truth online, for example, we might instead use the online environment to pursue other values: psychological reassurance, identity reinforcement, tribalism. Second, by increasing the cost of attaining the value we might, perversely, start to value it even more once obtained. If the truth is hard to find, it is all the more precious when we do find it and so there might be a special virtue or moral excellence associated with those that work hard to obtain true beliefs. We will not belabour the truth example here since we have published a longer case study analysis of it (Danaher and Sætra 2022). Interested readers can consult that case study for more detailed information on the nature of the value of truth and the mechanisms at play. The important point for now is simply that technology doesn’t always reduce costs; it sometimes increases them and this can have important moral effects.

This limits the analysis of technology to its effect on values. What about the impact of technology on our capacity to follow moral rules (rights and wrongs)? A similar dynamic plays out there. There is an easy and attractive way to think about it. Social scientists have long thought about moral norms and rules in terms of cooperative games (for example, see the literature review in Curry 2016; and Curry et al. 2020). Many moral rules can be thought of as formalised cooperative strategies in social games. The purpose of the moral rule is to increase the psychological and social costs of defection in a given social game. Technology can change this by either increasing or decreasing the costs of defection, or, if you want to look at it the other way around, by increasing or decreasing the benefits of cooperation (Morrow 2013). The change in payoffs might follow from a change of the choices we can make, but it can also simply come about through changes in the probability of being sanctioned as technology could enable better monitoring and tracking, or, conversely, make it easier to act without being monitored or tracked.

As an example of the first effect, consider the norm of politeness which is common in many societies. We think it is important to treat people decently and not be overly critical or insulting of them, even if some criticism is warranted. For deeply entrenched cultural, psychological, and biological reasons, we are often very reluctant to breach this norm. It makes us feel uncomfortable and we risk retaliation. But, as a cursory survey of Twitter or YouTube comments sections reveals, social media technology has drastically reduced the costs of defecting from this norm of politeness. The ability to provide anonymous online commentary creates a physical and mental distance that reduces the risks involved in attacking someone else in person, or backstabbing a person to someone else.

As an example of the second effect, consider the norm of religious conformity, which, again, is common in many societies. People often rebel against or reject conservative religious norms, particularly in their youth. Historically, it may have been possible for people to escape these norms by finding like-minded people in their local community or moving away from home. The rise of surveillance technology can make the costs associated with breaking from those various conservative norms much higher. Consider a girl from a religious community. Whereas she might have been able to break with certain norms in her private interactions with friends in a low-tech community, the ubiquitous presence of camera-equipped phones increases the risk that any transgression could be captured, shared and punished.

One hypothesis worth exploring is whether technology tends to reduce the upfront costs of certain choices while increasing their longer term costs (or unperceived externalities). To this point, we have been assuming that the effect of technology on decision-making costs is obvious and ascertainable. This may not always be the case. For example, the short-term advantages of carbon-based transport were immediately obvious to many people. But the longer-term costs, particularly to air quality and climate, were much less obvious. As we have become aware of those costs, the morality of excessive carbon-based transport has become questionable. Similarly, the short-term benefits of convenience and connection enabled by digital technology are obvious to most, but the longer-term costs in terms of lost privacy, data protection and algorithmic manipulation are less tangible and, perhaps, only now becoming apparent.

What’s the moral significance of this? Well, if our understanding of the costs and benefits of technology changes over time, then so too will the moral effect of those perceived costs and benefits. Actions that were initially thought to be permissible—for example, taking multiple foreign holidays via long-haul flights—may come to be thought impermissible, once their true costs are better known. We could also imagine this happening in the opposite direction. Where once the costs were perceived to be high, they are now perceived to be much lower, and this changes the permissibility or perceived value of using a particular technology. This might be happening now in relation to nuclear power: due to increased awareness of the threat of climate change people see it as a more viable option, despite its obvious risks.

### Mechanism 3—Technology Enables New Relationships

The third mechanism of change concerns the impact of technology on relationships. Much of human morality is relational in nature. Indeed, some might argue that morality is inherently relational, i.e. that it is only in deciding how we should relate to others that we generate moral beliefs and practices (Darwall 2006; Tomasello 2016). This may overstate the case. Some values might be largely individualistic and relevant in the absence of social relations. Still, no one would deny that relationships are a key part of morality. Some relationships are morally valuable and morally prized. Friendships and intimate partnerships, for example, are often said to be among the basic goods of human life. Other relationships are recognised as being instrumentally valuable (e.g. workplace relationships). Relationships are not always positive. Some relationships are sources of conflict and competition. It is no surprise then that many of our moral rules of conduct concern how we should relate to other people or ‘what we owe to one another’ to borrow the popular phrase (Scanlon 1998).

Technology can affect our relationships in several ways. These will crop up in our discussion of the next three mechanisms. The first way in which they can affect them is simply by enabling new relationships. This can happen in at least two distinct ways. First, certain technologies—transport and communications technologies being the most obvious—can give us access to new *human* relationship partners. Whereas once upon a time we might have been confined to our local villages or tribes for relationship partners, transport and communications technology allow us to connect to people in more distant locations. This widens the pool of potentially beneficial (and harmful) relationships. Second, certain technologies—AI and robotics being the most obvious—can create wholly new *non-human* relationship partners. This claim is more controversial, but if it does prove to be the case, it allows us to, potentially, move beyond the anthropocentric (and biocentric) nature of traditional moral beliefs and practices.

The moral effects of relationship expansion are multifarious. New relationships are potential sources of value and harm. We have to figure out which is the case. The wider pool of relationship partners is a boon, in one sense, because it allows us to access more potentially valuable relationships. A gay youth stuck in a conservative rural community can improve their lives by moving away to a city with a large gay community (for example). But there is a downside to this too. A wider pool of possible relationship partners, makes for more complicated and prolonged searches for ‘ideal’ partners, and an increased sense of moral regret over relationships that might have been. Relatedly, the broadening and more effective “clearing” of the partnership market might make it more difficult for some people to find partners. The wider pool enables some to find a better match, but it can also lead to a situation where some are effectively excluded from the dating market. This has, for example, been linked to the emergence of involuntary celibates (incels – see Beauchamp 2019; Ging 2019).

In addition to this, expanding the circle of potential relationship partners expands the scope of moral rules. We have to figure out what (if anything) we owe our new potential partners. A common effect of this is to expand the circle of moral concern, i.e. to cause us to apply the same rules and standards to distant others. This expansion of the moral circle is, indeed, one of the hallmarks of moral progress, according to one prominent school of thought (Singer 1981; Buchanan and Powell 2018; Anthis and Paez 2021).

The moral effect of technology on new relationships is complicated in a number of respects. It is not simply the case that technology adds new potential relationships to the mix. Technology also changes how we relate to others. Communications technology, for instance, gives us access to new relationship partners, but it mediates our relationships with them. Instead of connecting with them in flesh and blood, we connect with them through telephone lines, text messages, videolinks, and virtual reality platforms. These modes of communication strip away some of the traditional features of human-to-human relationships, while adding others. This can raise the question as to whether these technologically mediated relationships are as valuable as their traditional counterparts, or whether they instantiate a wholly new type of value. The longstanding debate about whether online friendships are as good as ‘real world’ friendships is an example of this (Turkle 2011, Fröding and Petersen 2012, Elder 2014). But irrespective of whether the relationships have the same value as their traditional counterparts, or involve a new type or form of value, the mediation can also give rise to new moral dilemmas and questions. For instance, is it okay to ‘ghost’ someone in online communications? Is it okay to use automated messenger services to communicate with loved ones (Selinger and Frischmann 2016; Danaher 2018)?

On top of this, in the case of AI and robotics, technology presents us with new relationship partners that may lack some of the attributes or properties associated with human relationship partners. This, again, raises the question as to whether the relationships we have with those technological artifacts holds the same kind of value as the relationships we have with humans, or whether they too could instantiate a new kind of value. Optimists argue that they can (e.g. Danaher 2019) or that even if this is not possible in the short-to-medium term, relationships with technological artifacts can have their own intrinsic and instrumental values (Ryland 2021). Pessimists take a different view. They argue that since technological artifacts will (for the time being or, potentially, forever) lack important properties of humans, the relationships we have with them will always be inferior. They will consequently embody different, more inferior, values. For example, Sætra argues that even if we can have loving relationships with robots, it will be a ‘deficient’ kind of love, not one based on mutuality and respect, but one based on power, convenience and control (Sætra 2021b). In addition to this, if technological relationship partners lack the properties of humans, questions are raised concerning our moral duties to them. Is it okay to abuse or mistreat a robot, for example, or should we apply a similar set of moral norms to our relationships with them (Danaher 2017; Petersen 2007)?

### Mechanism 4—Technology Changes the Burdens and Expectations Within Relationships

In addition to creating new relationships, technology can change the moral rules that apply within relationships. In particular, it can change the burdens and expectations within relationships, and the associated duties and privileges. This is a specific manifestation of the first and second mechanisms (changing options and changing costs) but one that applies specifically to the relationship context. Given the centrality of relationships to moral life, it is worth singling this out and discussing as a distinct mechanism.

One reason for this, is that this mechanism gets to the core of the relational nature of morality. Much of human activity is collaborative in nature. We work together to achieve common ends (albeit with plenty of conflict along the way). This is true in the family, at work and in politics. In order for this collaborative activity to work, people have to know what is expected of them and what they can expect from others. According to Michael Tomasello’s theory of moral origins, collaborative activity of this sort is the basis for our modern moral psychology (Tomasello 2016). When you and I work together toward a common end, I know what you expect of me and you know what I expect of you. These expectations form the basis of our role-related duties—the things we ought to do for one another. If we violate those expectations, we become targets of reactive moral attitudes: blame, shame, guilt and so on (Strawson 1962).

Technology often plays an important role in determining the role-related duties within relationships. Indeed, Tomasello’s theory of moral origins is, in part, a theory about the role of technology in shaping our role-related moral consciousness. One of the claims he makes is that the invention of projectile weapons (spears in particular) changed the kinds of animals that humans could hunt. It was now possible for a human to fell a large bison or deer with a well-aimed throw. But it was not easy to do this on your own. It was best if you worked in a team, with some team members chasing the animal into the open and others felling it with their weapons. Big game hunting thus became a cooperative endeavour, made possible by technology, with different people having different duties and responsibilities with respect to its common end. Domestic, labour-saving technologies provide another example. Historically, clothes washing and food preparation took up significant amounts of time.^2^ This labour was normally performed by women. The invention of washing machines, microwaves and pre-prepared meals changed this, at least to some extent. According to research by Greenwood, wide distribution of these technologies significantly reduced the amount of time spent on those tasks and made it possible for women to consider careers outside the home (Greenwood 2020). This, in turn, led to an expectation that they do so (and a sense that they were not pulling their weight if they did not). Nowadays, ensuring that women have this option is perceived as key for achieving gender equality, and the Sustainable Development Goal number 5, related to gender equality, even mentions enabling technologies that reduce women’s workload at home, as a key path towards the goal (United Nations 2015). There are, however, some sceptical counterpoints in this debate. Some argue that the invention of these technologies did not always reduce the amount of domestic labour performed by women (and it was women who were still expected to do these tasks). Instead, it increased the total amount of labour they were expected to perform and kept this labour within the home (Shehan and Moras 2006). So, for example, the availability of washing machines increased the expectation of having clean clothes and hence led to more clothes washing. We pass no judgment on which story is more accurate. The important point is that, either way, the technology is perceived to have changed the burdens and expectations within domestic relationships.

Consider another, more contemporary example: the effect of communications technologies on our workplace relations. The ‘always on’ nature of the internet, coupled with the everyday use of smart, internet-connected devices, has created an expectation of responsiveness in many workplaces. If you are sent an email, you are often expected to respond promptly. Indeed, you are often criticised (sometimes behind your back) if you do not. This creates a significant burden for many people. There is a backlash against this in recent times. Many people now add explicit sign-offs to their emails saying that they ‘do not expect people to respond outside of normal office hours’, and some countries have even made it illegal to send or expect “out of hours” responses to emails, at least for managers and employers (Morris 2017). The moral impact, as per usual, is complex and filtered through other moral beliefs and practices. The important point here is that the technology has had an impact on the burdens and expectations within workplace relations.

As hinted at above, the reason why technology has this impact on relationships is partly to do with the fact that technology adds options (mechanism 1) and changes costs (mechanism 2). By adding options and changing costs, we have to re-evaluate what it is that we can, and ought, to do for one another.

### Mechanism 5—Technologies Change the Balance of Power Within Relationships

Another way in which technology can affect relationships is by changing the balance of power within a relationship. Relationships are rarely perfectly equal. Oftentimes one person or one group has more power than the other. This has important moral effects. The powerful party typically derives more benefit (value) from the relationship and issues more moral demands of the other party. This can, in turn, generate considerable tension or instability in the social normative system. The weak may feel the need to rebel; the powerful may feel the need to reinforce their power, sometimes through draconian means.

Technology can either reduce or increase an imbalance of power. If it reduces a prior imbalance, this can have an equalising effect: the value of the relationship can be more equally shared and the moral duties and rights can become more equivalent. If it increases a prior imbalance, the powerful can extract more value from the relationship and impose further moral burdens and restrictions on the weaker party. It is also possible, of course, that technology could completely invert a prior imbalance so that the weak become the powerful, thereby enabling them to gain more from the relationship and impose heavier burdens (perhaps get their ‘revenge’) on the formerly powerful parties.

The industrial sociologist Stephen Barley has documented this effect of technology in several of his ethnographic studies (Barley 2020). Most of these focus on how technology changes the balance of power within workplace networks. One example is his study of the impact of the internet, and specifically internet sales, on the relationships between customers and car salespeople. As he points out, the traditional (roughly pre-2000) model of car sales (in the US) involved a hapless customer attending a car showroom. Once there, the customer would meet a salesperson. The salesperson would employ a number of sharp bargaining tactics to encourage the customer to sign up to purchasing a car on the day they entered the showroom. Based on his interviews, Barley found that customers were often frustrated by this process, many times regretting the purchasing decisions they made. He also noted that salespeople frequently lied in order to ingratiate themselves with customers and employed theatrical techniques to create a sense of urgency about the need to buy the car (Barley 2020, 56ff). In this traditional set-up, the salesperson had all the power. Customers rarely knew much about the cars they were buying, could not easily compare prices across dealers (or against list prices), and found it difficult to extract themselves from a negotiation after a certain point in time. That changed, quite dramatically, with the advent of internet and phone sales. Suddenly, the customer had more power. They could compare prices across dealers and they could easily extract themselves from unpleasant bargaining situations (e.g. by hanging up a phone). Barley found that, in response, the salespeople (operating mainly from behind desks and over the phone) adopted a more honest and less sharp bargaining style. In this case, the technology had an equalising effect on the relationship.

Another example of this trend is the impact that photography and audio-visual recording has had on the relationships between citizens and the state. On the one hand, these technologies have been leveraged by governments, enabling mass surveillance and control of the population. The classic example here might be the Stasi in East Germany, whose surveillance capability was dramatically represented in the 2006 movie *The Lives of Others*. More recently, of course, the digital surveillance powers of all governments were made obvious in the wake of Edward Snowden’s leak of information about the work of intelligence agencies in the US and Europe. On the other hand, the wide dispersal of recording devices through consumer markets has enabled ordinary citizens to speak truth to power, at least to some extent. They can do this by recording and sharing examples of police brutalities and human rights abuses. For example, the ease of capturing and spreading audio-visual material appears to have become an important weapon for Ukraine in their war against Russia, as they are able to spread awareness of what is happening in their country during the Russian invasion and gain unprecedented support and sympathy from countries have shown Ukraine is unprecedented. While this cannot surely not be attributed to media alone, it seems likely it plays an important role.

The advent of synthetic audio-visual materials (e.g. deepfakes) will, no doubt, also have effects on the balance of power. This technology allows actors to create and share hyper realistic fake audio-visual material. On the one hand, this could undermine those with power—it becomes harder for them to control the narrative in the wake of fake media—or it could empower them—allow them to create a propaganda record that matches their policy aims. At the same time, the desire to debunk fake media might empower a new technical elite that has the forensic know-how to sort the truth from fiction.

One final point worth noting is that when we talk about the impact of technology on relationships in general and power in particular, we should not limit our focus to person-to-person relationships. Technology can affect the relationships between different institutions (e.g. corporations, the state, civil society) and, even, more abstract entities (e.g. the public and private sectors). For instance, AI and big data have, quite clearly, changed the power relationship between the public and private sectors (with the public sector often reliant on proprietary private sector software). They have also affected the power relations between different regions (e.g. US vs. China).

### Mechanism 6—Technology Changes Moral Perception

The sixth, and from our perspective final, mechanism through which technology affects social morality is the most abstract: by changing our moral perception. In a sense, each of the previous mechanisms presupposes a change in moral perception. Technology may change options or decision-making costs but unless people are aware of those changes, their moral beliefs and practices will not change. So technology must change how we perceive the world in order to have any effect on morality. That seems to be trivially true. But there are also more subtle, effects on moral perception that can alter moral beliefs and practices. Technology can, for instance, change the modality or form of information/data we get from the world (radar images of approaching enemies, brain scans of locked-in patients, heart rate activity monitors and so on). This can give us information that is relevant to our moral decision-making. Knowing that someone’s heartbeat is irregular gives you information that could help to prevent a fatal heart attack and thus, arguably, imposes a new moral duty on you to intervene if you can.

In addition to providing new data, imagery and information, technology can also change mental models and metaphors. Much of human reasoning is analogical or model-based. In other words, we reason about the world through simplified mental models of how the world works, often building up these models by comparing them with other models. These models generate insight and practical guidance. Consider, for example, the simple supply-and-demand models employed by economists. These give some basic insights into how supply and demand are related to one another, and are often used to decide on practical policies, such as the wisdom of price floors or ceilings. We also employ analogical models in moral reasoning. Indeed, much of applied ethics proceeds from the analysis of abstract hypothetical cases. We use these cases to test moral intuitions and generate moral rules The use of ‘trolley’ thought experiments is one of the best examples of this.

One thing that technology can do is provide us with mental models and analogies for understanding the world. Sometimes it does this in a simple and direct way: by giving us new images which we use to interpret and understand the world. Sometimes it does it in a more abstract way. Gigerenzer and Goldstein (1996) point out that we often use our technological tools to develop theories of the world. They call this the ‘tools-to-theories’ heuristic. Prominent examples of this include the impact that the invention of the mechanical clock had on early physical theories (i.e. the model of the mechanical universe) and the impact that the computer has had on cognitive science (i.e. the computational model of the mind).

New mental models, heuristics and analogies can change our moral perceptions. One of the most famous illustrations of it can be found in Verbeek’s work on ‘hermeneutic’ moral mediation (which we see as an example of this mechanism at work). Verbeek argues the invention of obstetric ultrasound changed our moral perception of the foetus-in-utero. It gave us striking visual images of the foetus, presenting it to us as an independent biological being, not something hidden, abstract and biologically dependent. This imagery was not morally neutral: it changed our pre-existing moral concepts and understandings. As he puts it himself:

*“This technology is not merely a neutral interface between expecting parents and their unborn child: it helps to constitute what this child is for its parents and what the parents are in relation to their child. By revealing the unborn in terms of variables that mark its health condition, like the fold in the nape of the neck of the fetus, ultrasound ‘translates’ the unborn child into a possible patient, congenital diseases into preventable forms of suffering (provided that abortion is an available option) and expecting a child into choosing for a child, also after the conception.*” (Verbeek 2013, 77–78).

Verbeek would be the first to acknowledge that ultrasound did not change moral beliefs in a simple or linear way: it interacted with prior moral beliefs and commitments. If you previously thought the foetus was an unborn child deserving moral protection, the striking images of a miniature human being floating in utero probably reinforced this moral commitment. Contrariwise, if you thought the foetus did not deserve this protection, the effect might be different. As he suggests, it might be that you see the foetus as a medical patient that can be intervened and acted upon, perhaps to the point of preventing its existence for its own benefit.

Verbeek’s example is but one among many and involves a relatively simple and direct form of mental model-building. The ultrasound presents us with a new, previously hidden, image of the world that we interpret in a moralised way. More abstract mental models are also made possible by technology. Sætra’s discussion of ‘robotomorphy’ is a good example of this (Sætra 2021a). As he points out, the widespread use of rats as experimental models for humans has, arguably, resulted in the belief that humans are rat-like in important ways. This is an inverse form of anthropomorphism: instead of imposing human-like traits on the animal model we impose animal-like traits on humans. Sætra argues that the same thing can happen through the widespread use of robots in research and social life: we start to think of humans as being robot-like in crucial respects. The tool-to-theories heuristic, mentioned above, gets leveraged here. We use the tool to gain a deeper understanding of how humans work and interact with one another. But this also entails a risk that we reduce humanity to what can be reproduced in machines, leaving certain as of yet unobservable phenomena of potential importance, such as human experience, intentions, and emotions, out of the picture (Sætra 2021a).

This can have important moral effects in itself, as robotomorphy entails a change in how we perceive ourselves and others. How we treat others is premised on our perceptions of them, and if robotomorphy takes hold we might, for example, be more inclined to accept the algorithmic governance of humans through nudging, because any deviation from machine like rationality is, after all, merely a mistake and something to be eliminated (Sætra 2021a). One example of this line of thinking is found in Christian and Griffiths’ (2016) insistence and enthusiasm about the fact that the computational metaphor of the human mind “can utterly change the way we think about human rationality”. While the power of metaphors is certainly relevant today, we might also note that these issues were also a key concern for the progenitor of cybernetics, and Norbert Wiener’s (1950) *The Human Use of Human Beings* pre-empts many of the concerns discussed with relation to AI and new technologies today.

The moral effects of this last mechanism are hard to predict. New data and new mental models can have both modest and dramatic effects on morality. They may cause us to see a moral value that was previously hidden, or appreciate moral costs and benefits that were previously obscure; conversely they may hide moral values and costs that were once obvious (e.g. killing at a distance using an aerial drone vs. killing up close using a hand-held weapon). This may lead to a reprioritisation of values. For example, seeing data on climate change can change our perception of the value of the natural world. Imagine what might happen if we could develop brain-to-brain communication technology that allowed us to experience the pain of non-human animals. This can, in turn, affect moral rules: actions we once thought were permissible become clearly unacceptable and vice versa.

**Interim summary**: The six mechanisms we have described can be divided up into three main categories: decisional, relational and perceptual. In other words, technology can change morality by changing how we make decisions, how we relate to others and how we understand and perceive the world. The table below summarises the discussion to this point.

**Table:** 

Mechanisms of Technomoral Change						
Type	Decisional		Relational			Perceptual
**Mechanism**	Changes Option sets – typically by adding them, sometimes taking them away	Changes decision-making costs and benefits	Enables new relationships, both human and non-human	Changes burdens and expectations within relationships	Changes balance of power	Provides new information, data, mental models and metaphors
**Example**	Mechanical ventilation: adds option of maintaining organs after brain death	Effective contraception: reduces risks of unwanted pregnancy.	Digital communication: enables connections with distant others in mediated form. Robots/AI: create new potential relationship partners	Always-on communication, e.g. mobile phone, email, texting etc. – changes the expectation of availability and responsiveness	Audiovisual recording: enables mass surveillance and control; allows ordinary citizens to speak truth to power	Social robots and computational agents: encourages us to see humans as robot-like in certain key respects
**Moral Effect**	Raises new dilemmas; generates new moral rules (e.g. permissibility of using mechanical ventilation to enable organ donation	Makes values more or less accessible; generates corresponding duties and permissions, (e.g. permissibility of pre-marital sex)	Adds valuable but potentially different relationships; expands the moral circle, (e.g. robot lovers/friends allows to access some goods (companionship; pleasure) but not others (mutuality; respect))	Generates new moral duties and rights, (e.g. right to ‘switch off’ or duty to respond)	Redistributes values and goods, redistributes rights and duties (e.g. duty of transparency in response to increased scrutiny from citizenship)	Changes how we perceive the value of certain activities, events and states of affairs (e.g. human irrationality is a flaw that needs to be wiped out)

## Layered Mechanisms, Interactive Mechanisms, and Higher Order Effects

The preceding six mechanisms were discretely and neatly described. The practical reality of moral change is likely to be more complex. This is for a variety of reasons. We will discuss four here: (i) the moral effects can manifest at different *levels* (ii) the mechanisms can *layer* on top of one another; (iii) there can be *interactive* effects between different technologies; and (iv) technologies can have a *second* and *third* order moral effects. Let’s go through each of these in more detail.

First, consider the different levels at which moral effects can arise. We can distinguish between *micro*, *meso* and *macro* levels, although these designations are, of course, fuzzy. Some moral changes first occur in individuals, as they start thinking differently about what is right, wrong, valuable, not valuable and so on. This is the micro level. Other changes occur in, for example, organizations, as technology leads to changes in metaphors and the logic by which they approach human beings, leading to new forms of organization which consequently change norms and behaviour. This is the meso level. Finally, the macro level could relate to changes in power relations, and particularly how constitutive power—the power to change what people are and become—relates to technology (Sattarov 2019).

If we take, for example, the introduction of love and sex robots, we see that the implications are quite different at the different levels. At the micro level, individuals will experience an increased or decreased ability to find loving – or love-like – relationships, and these effects are both direct and important for the persons in question. At the meso level, such technologies might have different effects for different *kinds* of people, as mentioned in relation to how some become able to more effectively find partners while others are involuntarily excluded from the dating market. Such differences between groups are important, and provide the grounds for a diverse set of morally important consequences. At the macro level we find yet other potential consequences. What if, for example, people will in the future be more inclined to live in love-like relationships with machines? Issues of family policy and procreation might experience important shifts as a consequence, and this is likely to require political action that directly affects morality (Sætra 2021b).

Second, consider the ways in which mechanisms can layer on top of one another. Any one technology could, in principle, implicate each of the six mechanisms described in the preceding sections. For example, social-media enabled smartphones implicate several mechanisms of moral change at the same time. As noted, they add options to our lives: the option of capturing, archiving and sharing daily experiences. They lower the costs of connectivity and engagement with others. They enable new relationships with distant others – and even with the devices themselves. They affect the burdens and expectations within relationships, e.g. by increasing demand for responsivity and availability. They change the balance of power by giving us the ability to record information or send anonymous criticisms. And they can change the mental models we use in our daily lives: instead of seeing daily experiences as things that should be enjoyed in their own right, we see them as events that can be captured and monetised, or otherwise leveraged to enhance our social reputations. This new mental model affects the values and norms we adopt in our daily lives.

Third, consider the possible interactive effects of technologies. Two different technologies could pull us in opposite moral directions. For example, the automobile could be said to give us the option of driving while drunk; the alcohol interlock takes it away. Different technologies could also compound and reinforce the same moral effect: the internet, the camera-enabled smartphone, social media applications and machine learning, for example, all tend to put pressure on the value of privacy. Furthermore, a single technology could have different effects in different domains, leading to complex interactive moral effects. In an analysis of how AI relates to the Sustainable Development Goals, Sætra (2022) argues that the implications of AI are ripe with such interactive effects. For example, AI based surveillance can potentially make communities safer and help combat crime, while at the same time producing discriminatory effects. This might promote certain kinds of behaviour, but it can also end up shaping our expectations of others. The better technology approaches perfection in monitoring behaviour, for example, the less *trust* and faith in other people are required (Danaher and Sætra 2022). At the same time, such technologies have implications for privacy and our valuation of it.

Fourth, and finally, consider the possible second and third order moral effects of technology. What we mean here is that a particular technology might first affect our moral decision-making in one domain, but then have spillover effects in others. This can happen for several reasons. Technologies often have a primary use case but then get co-opted for different purposes. This leads to unanticipated downstream effects. Furthermore, by changing the decision-making calculus in one area, we can end up changing it in another, related, area. One good example of this is Adshade’s analysis of the impact of contraception on the permissibility (or social acceptability) of having a child outside of wedlock (Adshade 2013). As noted already, several analyses suggest that cheap and effective contraception largely eliminated the taboo of extra-marital sex. Adshade argues that some of the people that took advantage of this new social norm either had uncontracepted sex or experienced contraceptive failure. They, consequently, ended up having children out of wedlock (ignoring, for now, the effects of legalised abortion). This, somewhat ironically and paradoxically, reduced the stigma associated with having a child out of wedlock (the practice became more normalised and socially acceptable). The second-order effect (reducing the taboo of having a child out of wedlock) was an unanticipated consequence of the first-order effect (reducing the taboo of having sex).

Paying attention to these four complicating factors can enrich the mechanistic analysis of how technology changes morality.

## Conclusion

A concluding summary seems somewhat superfluous. This paper has adopted a simple structure. We have described six mechanisms through which technology can change social morality. We have also considered the complex layered, interactive and second (and third…) order effects of these mechanisms. What might be worth re-emphasising by way of conclusion is the intended purpose of this paper. We have not set out to defend any particular mechanism of technomoral change as being more important than any other. We have, rather, attempted to provide a reasonably comprehensive and synoptic account of these mechanisms. This helps to unify and explain the existing literature on technology and moral change. It also provides a framework for engineers, technologists, policy-makers and activists who might be concerned about the moral impact of technology.

## Data Availability

Not applicable.

## Footnotes

1 The Good vs. the Right distinction is common in moral philosophy. We take it from W.D. Ross’s classic statement (Ross 1930).

2 For a particularly compelling description of this, in a rural, pre-electrification, community, see Robert A Caro’s first volume in his biography of Lyndon B Johnson (*The Years of Lyndon B Johnson: The Path to Power*) and the chapter entitled “The Sad Irons”.
